# Defining a threshold for safe surgical management of vena cava thrombus in renal cell carcinoma patients: evidence from German total population data with 3,700 cases from 2006 to 2020

**DOI:** 10.1007/s00345-024-05360-z

**Published:** 2024-11-29

**Authors:** Thomas Martin, Johannes Huber, Rainer Koch, Marius Butea-Bocu, Lennard Haak, Luka Flegar, Matthias Giese, Fabian Kormann, Cem Aksoy, Aristeidis Zacharis, Christer Groeben

**Affiliations:** 1 Department of Urology, Philipps-University Marburg, Marburg, Germany; 2 Department of Urology, University hospital of Marburg, Baldingerstrasse, D-35043 Marburg, Germany

**Keywords:** Renal cell carcinoma, Tumor thrombus, Mortality, Hospital caseload, Health services research

## Abstract

**Purpose:**

The management of inferior vena cava (IVC) tumor thrombus in patients with renal cell carcinoma (RCC) is among the most challenging surgical procedures. We aimed to define a minimum annual caseload for sufficient expertise.

**Methods:**

We identified all cases with RCC, nephrectomy, and IVC procedures in the Federal Statistical Office billing database (2006–2020). We defined annual hospital caseload categories as low (< 4 cases), medium (4–9 cases) and high (> 9 cases) volume. Logistic multivariate models identified mortality-related factors. In addition, we analyzed data on tumor stage distribution from German cancer registries.

**Results:**

We recorded 3,700 nephrectomies with IVC-tumor resection with stable annual case number of 247 mean. This correlated with a stable incidence of T3b/c RCC. Patient age was 66 ± 14 years. Of all cases, 56% occurred in low, 30% in medium, and 14% in high volume clinics without a significant trend towards centralization. The overall in-hospital mortality rate was 5.8% and the transfusion rate 72%. An annual caseload of 8 showed to be a significant cut-off for mortality with 6.2% at < 8 cases and 2.8% for > = 8 cases annually (*p* < 0.001). Multivariate analysis revealed patient age (OR 6.4 for octogenerians) ventilation time (OR 14.3 for > 24 h) and hospital caseload (OR 2.6) as the most important risk factors for in-hospital mortality.

**Conclusion:**

Our results show a negative correlation of annual caseload and mortality for this procedure. A minimum number of 8 procedures per year seems reasonable for the successful management of IVC tumor thrombus with significantly lower mortality.

**Supplementary Information:**

The online version contains supplementary material available at 10.1007/s00345-024-05360-z.

## Background

For non-metastatic advanced renal cell carcinoma (RCC) with tumor thrombus formation extending into the vena cava, radical nephrectomy (RN) with cavotomy and thrombus extirpation is currently the only curative treatment [1–3]. Depending on the extent of the thrombus, this may even require opening the right cardiac atrium and thus the use of a temporary extracorporeal circuit. If parts of the wall of the vena cava have to be resected, replacement of the vena cava with alloplastic material is usually required [3–5]. This suggest that performing such a procedure requires an experienced surgical team with established interdisciplinary structures as well as appropriately equipped intensive care units.

Furthermore, due to the increased risk of extensive bleeding or extensive pulmonary embolism, this procedure also represents one of the uro-oncological interventions with the highest mortality and complication rates [6, 7]. Despite this obvious need for treatment in high-volume centers or specialists with appropriate surgical experience, no official guideline recommendations or awareness exists for this situation. Furthermore, no current evidence exists on treatment patterns or trends for the distribution of patients in Germany [2].

Therefore, the aim of our study was to examine the development of treatment patterns in the management of a renal cell carcinoma tumor thrombus (RCCTT) in the last two decades and assess possible impact factors on in-hospital mortality.

## Methods

### Data sources

The nationwide hospital billing database of the German Federal Statistical Office was used as primary data source. We described the methods of data extraction and cohort identification in previous publications [8]. The database is coded using the 10th revision of the International Classification of Disease (ICD-10) for diagnoses and German classification of operations and procedures (OPS)) being transferred annually by all German hospitals to the Federal Statistical Office.

We included all cases with a diagnosis of renal cancer (ICD-10: C.64) in combination with a RN (OPS-code 5-554) and incision, embolectomy and thrombectomy (5-380), resection with reanastomosis (5-382) or resection and replacement (interposition) of (parts of) (5-383) the vena cava. The surgical approach was grouped as open-lumbar, open-transabdominal, thoracoabdominal or laparoscopic. Factors for stratification were age, gender and hospital characteristics (i.e. teaching status, hospital size, and academic status). Annual hospital caseload categories were defined as low (< 4), medium (4–9), and high (≥ 10) according to our previous work on rare entities or procedures [9, 10].

We supplemented the nationwide incidence of renal cancer from the German “National Centre for Cancer Registry Data” at the Robert Koch-Institute to calculate tumor stage distribution and development over time [11]. The data included from the “National Centre for Cancer Registry Data” is presented in absolute numbers and age standardized incidence rates (old European standard population).

### Statistics

We extracted the absolute number of cases and calculated rates for mortality and blood transfusion as well as length of hospital stay (LOS). Results were stratified for age, gender, surgical approach, and annual hospital caseload. We applied linear models for LOS, and logistic models for in-hospital mortality. To detect trends over time, linear regression was implemented. We compared rates, means and trends using Wald-tests. For determination of the optimal Cut-Off-Value for annual caseload to reduce mortality, Chi-Square tests were applied. A value level of *p* < 0.05 was regarded as significant. We used SAS 9.4 (SAS Institute GmbH, Heidelberg, Germany) for all analyses.

## Results

### Epidemiology

According to the cancer registry data the absolute incidence of renal cancer in Germany remained stable from 14,813 cases in 2006 to 14,087 in 2019 (*p* = 0,42 for trend). However, the age standardized ratio (ASR) showed a declining tendency from 9.1 (2006) to 7.5/100,000 (2019) (*p* = 0.05). The mean age of renal cancer patients at diagnosis remained stable at 67.7 ± 13.0. Overall, 7.1% of all patients presented with lymph node and 14.1% with distant metastasis at primary diagnosis. The share of patients diagnosed with stage T3b/c-disease decreased from 2.0% (2010) to 1.3% (2020) (*p* < 0.001). Of those 34.1% were female.

### Surgical management of a RCCTT

**Fig. 1:**
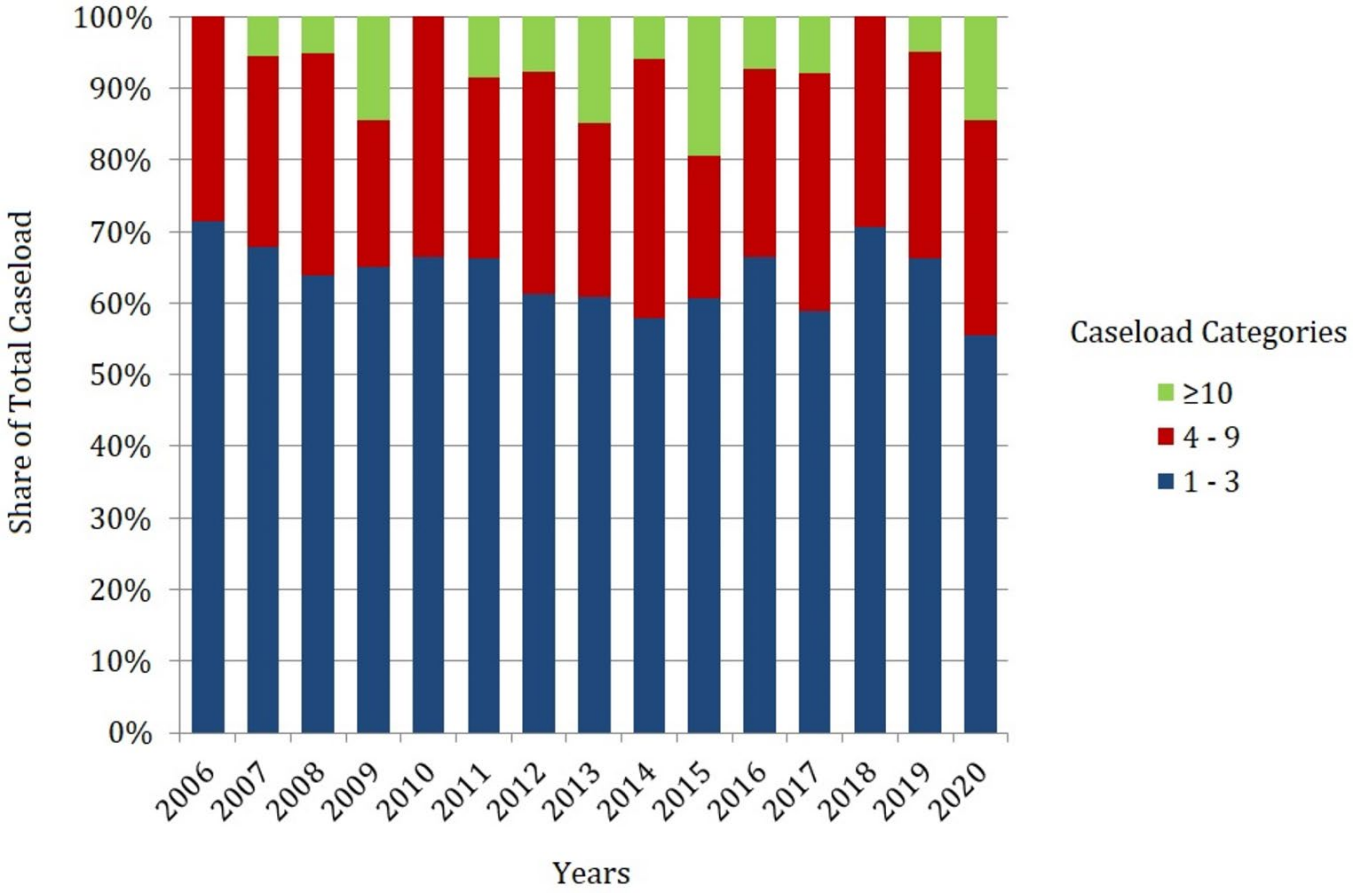
Development of Caseload distribution among caseload categories per hospital and year for surgical management of RCCTT in Germany

A total of 3,700 cases of surgery for RCC with resection of a RCCTT were extracted from the billing-database with stable annual numbers at 181 in 2006 and 220 in 2020 (*p* = 0,13 for trend). The average number of hospitals was 123.7 per year. Patient characteristics and patterns of care for surgery for RCCTT are presented in Table 1. Mean patient age was 66.1 ± 14.0 (SD) years with a mean share of 34.4% female patients. Shares of the transperitoneal approach increased from 72.5 to 82.2% (*p* < 0.001) while thoracoabdominal intervention decreased from 13.5 to 4.2% (*p* < 0.001) and lumbar nephrectomy with thrombus resection remained stable at approximately 13.4%. Extracorporeal circulation had to be implemented in 5.6% of cases. Figure 1 shows the volume of surgery for renal cancer with RCCTT according to the hospital caseload category. The share of patients treated in hospitals with > 3 cases per year seemed to increase without statistical significance from 28.5% in 2006 to 44.5% in 2020 (*p* = 0.10).

**Table 1:** Patient characteristics and patterns of care of patients with surgical treatment for RCCTT in Germany (2006–2020)

		Nephrectomy + RCCTT-Resection
Total number of cases		3,700
Age (years)		66.1 ± 14.0 (SD)
Age Group (years)	**< 50**	326 (8.8%)
	**50–59**	581 (15.7%)
	**60–69**	1084 (29.3%)
	**70–79**	1299 (35.1%)
	**> 79**	407 (11.0%)
Gender female		1,271 (34.2%)
Annual Hospital Caseload	1–3	2,355 (63.7%)
	4–9	1,044 (28.2%)
	> 9	301(8.1%)
Teaching Status	academic	1,281 (34.6%)
Primary Department	Urology	3,419 (92.4%)
Size of Hospital	< 300 beds	124 (3.4%)
	301–800 beds	796 (21.5%)
	> 800 beds	1,707 (46.1%)
	missing	1,073 (29.0%)
Surrounding City Size (Inhabitants)	< 20,000	113 (3.4%)
	20,001–100,000	675 (18.2%)
	100,001–500,000	1,022 (27.6%)
	> 500,000	814 (22.0%)
	missing	1,073 (29.0%)

### Perioperative outcome

The overall rate of blood transfusion was 71.9% of cases with a continuous decrease from 77.8 to 66.1% over the study period (trend *p* = 0.002). The transfusion rate concerning surgical approach was 73.2% for transperitoneal, 82.3% for thoracoabdominal and 58.8% for lumbar nephrectomy (*p* < 0.001). Higher hospital caseload was associated with slightly higher transfusion rates (< 4 cases 71.3% vs. 4–9 cases 76.3% vs. >9 cases 75.5%; *p* = 0.023).

The overall LOS decreased from 18.2 ± 10.2 to 15.6 ± 13.8 days (*p* = 0.001). LOS was longer for the thoracoabdominal approach with 20.3 ± 14.6 versus 17.5 ± 12.2 (SD) (transabdominal) and 15.5 ± 10.1 days for the lumbar nephrectomy (*p* < 0.001). High volume hospitals presented shorter LOS (< 4 cases 17.5 ± 12.1 vs. 4–9 cases 18.1 ± 13.0 vs. >9 cases 15.9 ± 10.0 days *p* = 0.028, < 4 vs. >9 cases). Multivariate analysis (Table 2) indicated ventilation time to be the most important factor for LOS followed by annual caseload and surgical approach.

**Table 2:** Multivariate models for influential factors on mortality and LOS for surgical treatment of RCCTT in Germany from 2006–2020. (CI = confidence interval; ref. = reference for model, bold values = statistically significant)

		Mortality	*p*	Length of stay		*p*
Variable		**Odds Ratio**		**days**		
		(95% CI)		(95% CI)		
				**Intercept Value**	**15.78 (14.19–17.37)**	-
Category of annual caseload per hospital	1–3	**2.62 (1.30–5.32)**	**0.007**	**1.63 (0.32–2.95)**		**0.015**
	4–9	**2.98 (1.48–5.98)**	**0.002**	0.98 (-0.33–2.29)		0.144
	> 9 (Ref.)	1	**-**	0		-
Gender	Male (Ref.)	1	-	0		-
	Female	**0.63 (0.45–0.89)**	**0.009**	**0.90 (0.13–1.67)**		**0.022**
Hospital teaching status	Academic (Ref.)	1	-	0		-
	Non-Academic	1.48 (0.88–2.49)	0.139	0.23 (-1.05–1.50)		0.725
Age Group (years)	< 50 (Ref.)	1	-	0		-
	50–59	0.76 (0.32–1.79)	0.528	**-1.54 (-3.08 - -0.01)**		**0.048**
	60–69	1.18 (0.58–2.40)	0.651	-1.13 (-2.53 - -0.27)		0.113
	70–79	**2.14 (1.08–4.23)**	**0.029**	-0.53 (-1.91–0.84)		0.446
	> 79	**5.81 (2.81–12.00)**	**< 0.001**	0.16 (-1.50–1.82)		0.851
Size of Hospital (Beds)	Small (< 300)	0.84 (0.52–1.35)	0.468	**-2.24 (-3.44 - -1.04)**		**< 0.001**
	Medium (300–800)	0.90 (0.55–1.47)	0.669	-1.23 (-2.47–0.01)		0.053
	Large (> 800; Ref.)	1	-	0		-
Surgical Approach	Abdominal (Ref.)	1	-	0		-
	Thoracoabdominal	0.75 (0.46–1.23)	0.250	0.78 (-0.46–2.02)		0.218
	Lumbar	0.78 (0.46–1.30)	0.338	**-1.53 (-2.61 - -0.45)**		**0.006**
	Other (Laparoscopic)	0.78 (0.08–7.57)	0.828	-3.13 (-7.66–1.40)		0.175
Ventilation Time (hours)	0–12 (Ref.)	1	-	0		-
	13–24	**3.56 (1.47–8.63)**	**0.005**	**7.04 (4.53–9.56)**		**< 0.001**
	25–120	**9.70 (6.63–14.19)**	**< 0.001**	**6.86 (5.56–8.15)**		**< 0.001**
	> 120	**28.91 (19.26–43.39)**	**< 0.001**	**22.82 (21.14–24.49)**		**< 0.001**

**Fig. 2:**
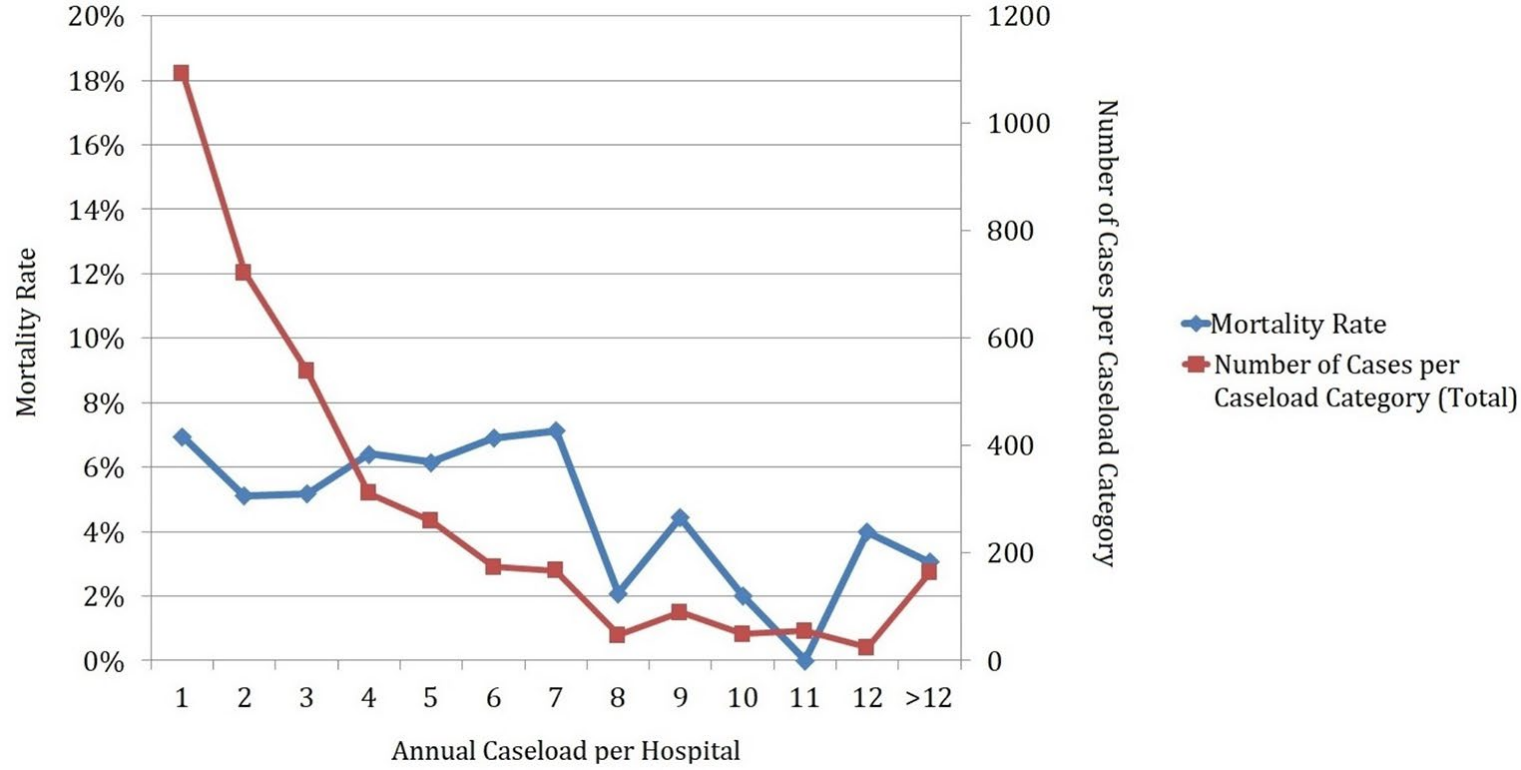
Relation of annual caseload per hospital and mortality for surgical management of RCCTT in Germany

Total in-hospital mortality was 5.8% (*n* = 213). Mortality rates differed widely throughout the years without tendency with a minimum of 3.4% in 2020 and a maximum of 8.7% in 2013. On univariate analysis, higher annual caseloads were associated with lower mortality (< 4 cases 6.0% vs. 4–9 cases 6.2% vs. >9 cases 2.3%; for low vs. high *p* < 0.001). On multivariate analysis (Table 2), the most influential factor for in-hospital mortality was ventilation time followed by age, annual hospital caseload and gender. Figure 2 demonstrates the correlation of in-hospital mortality and surgical experience represented by annual hospital caseload of RCCTT management. For patients receiving surgery in hospitals with an annual caseload of < 8 per year mortality rate was 6.2% compared to 2.8% in hospitals with ≥ 8 (*p* < 0.001). We demonstrated the comparison of different cut-off-values in Supplementary Table 1.

## Discussion

In our population-based analysis, an annual caseload of surgery for RCC with RCCTT was associated with reduced mortality and LOS, while shares of total caseload for high volume hospitals are slowly but steadily increasing. A minimum number of 8 procedures per year a reduction of mortality rate from 6.2 to 2.8%. Meanwhile, tumor incidence and total caseload numbers remained stable throughout the study period.

### Correlation between caseload and mortality

In our analysis, the most influential factor for in-hospital mortality was ventilation time followed by age, annual hospital caseload, and gender. Likewise, ventilation time was the most important factor for LOS followed by annual caseload. The association with ventilation time seems self-evident, since survival probability decreases continuously with the time under ventilation in an Intensive care unit [12]. The correlation of higher annual caseload of hospitals and improved outcomes (e.g. mortality, complications) for complex oncologic surgery (e.g. cystectomy, retroperitoneal lymphnode dissection, esophageal surgery) has been shown numerous times in different patient collectives or population based analysis [13–15]. Likewise, surgery for a RCCTT is a technically challenging procedure consuming relevant resources. Not only surgical experience but also providing an interdisciplinary team for selected cases (e.g. major vascular or thoracic surgery) is necessary [3–5, 16]. This high degree of surgical experience depends on performing a sufficient annual caseload. At the same time high caseload is a prerequisite for successful surgical education. In our multivariate models (Table 2) we were able to show the strong correlation between annual caseload and outcome for this procedure with odds for mortality increasing to 2.6 and the LOS increasing by 1.6 days in hospitals with low caseload compared to high volume centers. For extensive but rather rare surgical procedures like treatment for RCCTT minimum annual caseloads of 8 cases seem reasonable to achieve adequate outcomes when compared to other challenging procedures like radical cystectomy [13, 17] or retroperitoneal lymphnode dissection [10]. We consequently calculated the optimal caseload cut-off for improved outcome for this procedure (Supplementary-Table 1) revealing a caseload of at least 8 per year reducing the mortality to roughly one third. This number correlates well to comparable tumor surgery in the current literature and in our former projects [9, 10, 14, 15, 18].

In our analysis, incidental findings showed higher transfusion rates for centers with an increased number of cases. This is consistent with previous results from our study group [10, 14]. We suspect that this is due to the referral of more complex and demanding cases with a consecutively higher probability of transfusion to centers with a larger number of cases and higher surgical expertise.

### Possible measures of health policy making

No significant trend towards centralization could be detected throughout the study period (Fig. 1). Since 2004 German health care policy has begun to introduce minimum caseload requirements (e.g. for pancreatic surgery, hip replacement). To date however, without urologic oncologic surgery [19]. Based on our results and the evidence in current literature it seems reasonable to include resection of a RCCTT and other extensive procedures such as radical cystectomy as well. By denying financial remuneration for the respective interventions to hospitals that fell short of the required minimum volumes, German health policy aimed to exert centralizing pressure on the entire hospital landscape. However, to date no relevant effect of these measures could be recorded - possibly due to broad exemption rules allowing for repetitive avoiding of the strict regulations [19, 20]. Examples from other health care systems show that centralization is feasible for the benefit of patients [21–24].

### Population and epidemiology

The total annual numbers of cases with surgical treatment of RCCTT remained fairly stable around a number of 200 per year. This trend is mirrored by a rather steady overall incidence of RCC around 14,500 per year in Germany [25]. Shares of pT3b/c between 2.0% and 1.3% with respective yearly incidence numbers between 182 and 300 show that the share of patients with a RCCTT receiving surgical treatment for their disease with approximately 80 to 100% is very high. This could be mainly due to a timely need for surgical management to prevent symptoms or death of the patient leaving no alternative option for cure [3, 5, 16]. The overall patient cohort regarding age and gender is comparable to reported cohorts in other western health care systems such as the US [26].

### Limitations and strengths

Our study is the first to analyze treatment patterns of surgical management for RCCTT and demonstrate the correlation of caseload and in-hospital mortality in Germany using total population data covering 15 years. In order to empirically define a minimum number of cases by means of a hard endpoint (mortality) for this procedure, this group offers the largest possible number of cases. Furthermore, the almost exact consistency between the cancer registry data and the German Billing Database shows that the results are highly representative. The main study limitations can be derived from the nature of the data itself. German hospital billing data does not include detailed information on tumor and patient characteristics and due to data protection regulations single patients or institutions may not be identified and checked for correct data entry. All outcomes are limited to the in-patient stay. Finally, while the dataset of the German Cancer Registries encompasses all conceivable cases documented in the registry, it does not account for undocumented instances or incomplete follow-up information. Given the extensive caseload numbers for this rare procedure small irregularities appear to be negligible. However, the principal risk of systematic bias has to be kept in mind when interpreting the results.

## Conclusions

High annual caseload of surgery for RCC with RCCTT was associated with reduced mortality and LOS, while no significant trend towards centralization could be detected. Multivariate analysis also revealed patient age and ventilation time as important risk factors for in-hospital mortality. A minimum number of 8 procedures per year seems reasonable for the successful surgical management of RCCTT with significantly lower mortality. Minimal caseload requirements could be implemented to support centralization of surgical RCCTT management.

## Electronic supplementary material

Below is the link to the electronic supplementary material.

Supplementary Material 1

## Data Availability

The data that support the findings of this study are not publicly available due to anonymization regulations of the German Federal Office of Statistics. Aqcuired results and calculations are available from the corresponding author [CG] upon reasonable request.

## Abbreviations

RCC: Renal cell carcinoma
IVC: Inferior Vena Cava
RCCTT: Renal cell carcinoma tumor thrombus
SD: Standard Deviation
RN: Radical nephrectomy
LOS: Length of hospital stay
ICD: International classification of diseases
OPS: Operationen und Prozedurenschluessel = Classification of Operations and procedures
