# Supplementary material for: Defining a threshold for safe surgical management of vena cava thrombus in renal cell carcinoma patients: evidence from German total population data with 3,700 cases from 2006 to 2020
Source: World J Urol. 2024 Nov 29;43(1):1. doi: 10.1007/s00345-024-05360-z (PMC11606981; doi:10.1007/s00345-024-05360-z)
Supplement: Supplementary file 1 — Supplementary Material 1 [file 345_2024_5360_MOESM1_ESM.docx]

**Supplementary Table 1:** Comparison of cut-off values for annual hospital caseload, compared to the resulting mortality rate.

| **Annual hospital Caseload Cut-off Value** | **Mortality rate above Cut-off Value** | **Mortality rate below Cut-off Value** | **Relative risk reduction** | **p-Value** |
| --- | --- | --- | --- | --- |
| 6 | 6.05% | 5.90% | 2.41% | 0.197 |
| 7 | 6.09% | 5.50% | 9.68% | 0.063 |
| 8 | 6.15% | 2.78% | 54.82% | 0.007 |
| 9 | 6.05% | 2.86% | 52.62% | 0.014 |
| 10 | 5.96% | 2.38% | 60.04% | 0.012 |
| 11 | 5.99% | 2.46% | 58.93% | 0.027 |
